# The Eastern Door Center: re-balancing the wheel–a Two-Eyed Seeing approach to FASD and other disorders related to transgenerational adversity

**DOI:** 10.3389/fsoc.2023.910153

**Published:** 2023-05-05

**Authors:** Lori Vitale Cox

**Affiliations:** University of British Columbia, Vancouver, BC, Canada

**Keywords:** Fetal Alcohol Spectrum Disorder (FASD), Two-Eyed Seeing (TES), Indigenous health, transgenerational adversity, colonial trauma, childhood adversity

## Abstract

In 2015, the Canadian Truth and Reconciliation Commission (TRC) called for immediate action to address the lack of access to health services for Fetal Alcohol Spectrum Disorder (FASD) in Indigenous communities. They called for the provision of culturally safe, community-based, FASD diagnostic, intervention and prevention services. FASD is a neurodevelopmental condition that can affect all aspects of functioning. The term refers to a spectrum of conditions occurring as a result of prenatal alcohol exposure (PAE) and associated risk factors. PAE can affect both physical and mental health leading to problems with learning, memory, attention, language, social behavior, executive functioning, sleep, and affect regulation. According to Elders in Mi'kmaq First Nations (FN) communities, FASD is a condition that is rooted in transgenerational trauma and the loss of relationship to their land, their language and the traditional community culture. The Elsipogtog Eastern Door (ED) Center opened in 2006 to provide culturally informed diagnosis, intervention and prevention for FASD and related conditions. The ED was the first FASD diagnostic team in Atlantic Canada and it served as a demonstration model for the New Brunswick FASD Center of Excellence as well as for Indigenous communities regionally and nationally. In this article, we outline the history and evolution of the Eastern Door Center and its programs and describe some of the successes of this model as well as some of its limitations in practice.

## Introduction

The Eastern Door (ED) Center, located in an Indigenous community in Atlantic Canada, offers a model of health service delivery for conditions related to transgenerational trauma such as Fetal Alcohol Spectrum Disorder (FASD). It uses a Two-Eyed Seeing (TES) approach to FASD service delivery. TES is a concept developed by Elders Murdena and Albert Marshall: if one eye looks from a traditional perspective and the other from a scientific one there is more depth in perception (Marshall et al., 2009; Martin, 2012).

FASD is a neurodevelopmental condition that can affect all aspects of functioning. The term refers to a spectrum of conditions occuring as a result of prenatal alcohol exposure (PAE) (Astley, 2004; Chudley et al., 2005; Cook et al., 2016; Hoyme et al., 2016). PAE can affect both physical and mental health leading to problems with learning, memory, attention, language, social behavior, executive functioning, sleep, and affect regulation (Riley et al., 2003; American Psychiatric Association, 2013; Kable et al., 2016). PAE has neuropsychiatric consequences exerting stress on the developing fetus with activation of the hypothalamic, pituitary, adrenal (HPA) axis and dysregulation of cortisol and cytokine (Weinberg et al., 2008; Kobor and Weinberg, 2011). Fetal programming carries this dysregulation throughout an individual's lifetime and is associated with childhood affect regulation and adult depression and anxiety (Hellemans et al., 2010; Burgess and Moritz, 2020). Approximately 90% of individuals living with FASD have additional psychiatric co-morbidities (Streissguth and O'Malley, 2000).

The World Health Organization (WHO) recognizes FASD as a significant global public health problem (Lange et al., 2017). Conservative estimates of prevalence rates in WHO European regions, Canada and the US range from 2 to 5% (May et al., 2009, 2018; Popova et al., 2017, 2019a,b). South Africa has one of the highest FASD rates estimated at 11% (Lange et al., 2017). In some rural South African communities rates were as high as 31% with intergenerational nutritional trauma identified as a risk factor (May et al., 2022). Indigenous populations are among global subpopulations with estimated FASD prevalence rates 10–40 times higher than the general population (Popova et al., 2019a). Research in this area is critically limited, and can lack consideration of the broader social determinants of health (DOH) that influence substance use, mental health, and wellbeing (Tait, 2003; Di Pietro and Illes, 2014). Indigenous populations are at greater risk because of health inequalities in terms of DOH and transgenerational adversity (Barker, 1990; Gracey and King, 2009; Kobor and Weinberg, 2011; Wallack and Thornburg, 2016; Edwards, 2017; Marmot, 2017; Ciafrè et al., 2020). Risk factors for FASD are associated with poor nutrition, neglect, abuse, low socioeconomic status (SES) (Bingol et al., 1987; Abel and Hannigan, 1994; Abel, 1995; Kobor and Weinberg, 2011; May and Gossage, 2011). Adversity related to intergenerational and childhood trauma has been shown to affect the clinical expression of FASD as well as the secondary conditions associated with it. (Bingol et al., 1987; Streissguth et al., 1996; Koponen et al., 2009, 2013; Price et al., 2017; Mukherjee et al., 2019). However, the nature and extent of the relationship between trauma and PAE requires more research (Price, 2019).

FASD is a global problem and affects all races and social classes (EuFASD, 2022). The colonial process exacerbates both PAE and associated risk factors. In Canada as well as South Africa, alcohol has been used as a means of social control leading to patterns of binge drinking among women of gestational age (Daschuk, 2013; May et al., 2019). Overall rates of alcohol consumption among Canadian Indigenous women are reported as lower than in the general population, but binge drinking, associated with greater fetal risk, is reported to be more prevalent (Statistics Canada, 2001). Other risk factors are also more prevalent such as food insecurity and lack of access to prenatal care and other health services (Tait, 2003; Adelson, 2005; Waldram et al., 2006; King et al., 2009; Daschuk, 2013). Lack of access to FASD service delivery is exacerbated by these issues of social and economic marginalization as well as geographic isolation (Peadon et al., 2008; Clarren et al., 2011; Salmon and Clarren, 2011).

Before the ED Center opened in 2006 some specialists working in urban areas in the region offered diagnosis for only one FASD condition, Fetal Alcohol Syndrome (FAS). The more prevalent FASD conditions required the input of a multi-disciplinary team but few existed in Canada (Chudley et al., 2005; Cook et al., 2016).

In 2015, the Canadian Truth and Reconciliation Commission (TRC) called for immediate action to address the lack of access to health services for FASD in Indigenous communities. They called for the provision of culturally safe, community-based, FASD diagnosis intervention and prevention services [Truth Reconciliation Commission of Canada (TRC), 2015].

## The development if the Eastern Door Center model

The social and economic costs of FASD are high; costs are calculated to be approximately CA $24,000 per annum per individual diagnosed with FASD (Greenmyer et al., 2018). FASD is a lifelong disability which has engendered hesitancy among some health policy makers to fund FASD diagnosis and the disability accommodations that should follow. The cost of FASD service delivery may be considerably less than the cost of not providing services (Cox, 2015).

Without access to FASD diagnosis and intervention, individuals living with FASD may experience an array of secondary problems that compound the primary neurocognitive impairment. Adverse secondary outcomes relate to mental health, addictions, education and the conflict with the law, among other challenges (Streissguth and Kanter, 1997). These may be exacerbated by the effects of childhood or transgenerational adversity. Without diagnosis an FASD disability may be mistaken for criminality (Truth Reconciliation Commission of Canada (TRC), 2015). Up to 60% of people with FASD may come into conflict with the legal system (Streissguth and Kanter, 1997; Cox et al., 2008). Access to early diagnosis and provision of supports in school and the community are protective factors that lead to improved outcome (Streissguth et al., 1996; Cox et al., 2008).

There have been many community programs dealing with diverse elements of FASD service delivery but few are comprehensive (Place, 2007). FASD is complex condition and requires a multi-pronged approach (Flannigan et al., 2022). This is especially true in an Indigenous community impacted by effects of transgenerational adversity, marginalization, health inequalities, cultural stress, environmental adversity and the social stigma of FASD. There can be resistance in some Indigenous communities to FASD diagnosis where it may be perceived as labeling and part of the colonial process (Tait, 2003).

An Indigenous model of FASD service delivery rooted in the community context can provide a culturally safe space for disclosure and healing. The need to develop culturally and scientifically informed models that are cost-effective, comprehensive, community-based and replicable is recognized within and without Indigenous communities [Salmon and Clarren, 2011; Cox, 2015; Truth Reconciliation Commission of Canada (TRC), 2015].

The ED Center opened in 2006 to provide such a model. The ED was the first FASD diagnostic and intervention team in Atlantic Canada; it served as a demonstration model for the New Brunswick FASD Center of Excellence as well as for Indigenous communities regionally and nationally (Clairmont, 2010). Internationally, the ED has links with Indigenous community researchers in both Australia and New Zealand. In the Fitzroy Valley in Australia the Marulu strategy offers another Indigenous community model of FASD practice that is culturally rooted and safe (Fitzpatrick et al., 2012; Marulu Strategy, 2019).

## Community context

The ED is located in Elsipogtog, the largest Mi'kmaq First Nations community in New Brunswick, the second largest in the Atlantic Provinces. It has a population of approximately 3,800 people; more than 50% are youth. Rates of welfare and substance use are high as is food insecurity (Clairmont, 2003, 2010).

A youth suicide crisis in the community in the 1990's was the catalyst that precipitated the development of the Eastern Door Center fourteen years later. In response to the youth suicides the community gathered together for a week of mourning and healing—combining Mi'kmaq spirituality, Christianity and western psychotherapy (Royal Commission Report: Parliamentary Information and Research Services, 1995). The Royal Commission (1995) identified alcohol use, gas sniffing, mental health issues, family breakdown and unidentified learning disabilities as possible issues in the youth suicides. Elders in the community also identified loss of land and traditional language and culture as factors.

The Mi'kmaq people traditionally were hunters, fishers and gatherers with a primarily oral culture. With restricted access to the land came food insecurity and dependency. With loss of language transmission of traditional knowledge from one generation to the next was disrupted. Parents were compelled to send their children to the regional residential school in Shubenachadie Nova Scotia or to government “Day Schools”. Community Elders report that in these schools traditional culture was devalued and they were beaten for speaking the Mi'kmaq language (ED-CIHR Elders Focus Group, 2019).

The Mi'kmaq word Nogamag, literally “all my relations”, expresses a concept of relationship and connection that ties all things in this world and the spirit world together. The ties of Noegmag enable youth to connect to their spirits and make sense of their lives (Cox, 2015). These threads were torn with European settlement, the implementation of the reserve system, residential and day schooling and the gradual loss of access to land resources (McMillan, 2002).

### Community collaboration–integration of Mi'kmaq and Western healing traditions

**Figure 1:**
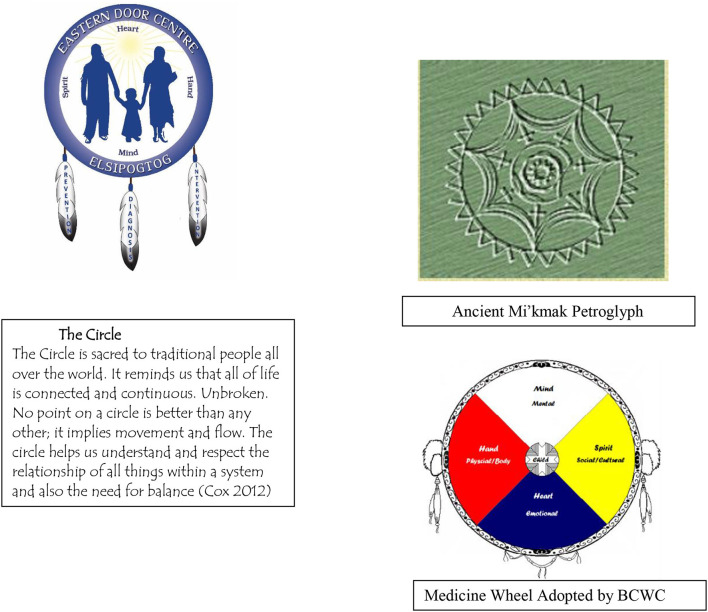
Mi'kmaq circle and Medicine Wheel.

In response to the community suicide crisis, an interagency community committee formed called the Big Cove Wellness Committee (BCWC). The BCWC adopted a Medicine Wheel (MW) framework with seven directions to support the integration of Mi'kmaq and Western healing traditions. The Mi'kmaq people used circle frameworks in their traditional practice but only now do they call them MW (Elder Joe John Sanipass, 2012) (Figure 1).

### School needs assessment

The BCWC lobbied Indian and Northern Affairs Canada (INAC) and received funding for two psychology positions, one in the school and one in the community. Despite this support the youth suicides and the turmoil in the school continued. Consequently there was rapid turnover in these positions. In 1997, a new school psychologist with a research background met with members of the BCWC about the on-going youth problems. The group decided that a comprehensive school needs assessment was necessary to identify unmet youth needs that might be affecting their wellbeing. Through participant action research (PAR), a series of community focus groups were held with teachers, parents, professionals, and Elders. Participants agreed youth in the community had complex social, emotional, mental, and physical needs interfering with their wellbeing. The object of the needs assessment inquiry would be to identify these needs more precisely in order to meet them (Cox, 2012).

Two screening tools were developed that attempted to integrate the community perspective in a medicine wheel framework. These tools were used to indicate children in the school who might have complex needs and to identify their particular areas of need. Parents and teachers both participated in the screening process as informants. With revisions these tools, the MW Teacher-Student Index Tool (MWSTI) and the MW Developmental History Interview (MWDH) are still used in the community. Mothers of all of the youth, identified by teachers as having learning and behavioral needs in school, disclosed their children had experienced high levels of developmental adversity- including food insecurity, family suicide, family mental health and addiction problems and pre-natal exposures to drugs, nicotine and alcohol (Cox, 2012). Despite research findings on the harms of PAE, clinical practice guidelines regarding alcohol use during pregnancy only came out in 2010. Local physicians told women in the community in the 1990's and early 2000's that mild to moderate drinking during pregnancy was safe. Knowledge and attitudes of many health professionals in the Atlantic regarding PAE and FASD was limited at the time (Tough et al., 2005). This may be the reason that mothers were willing to disclose prenatal alcohol use.

Parents hoped, at the time, that full medical assessments would identify underlying issues that then might be remediated. A developmental pediatrician held two clinics at the school assisted by the community nurse and the school psychologist (Cox, 2012). Children were then referred as needed for services or further assessment for conditions such as visual and hearing impairments, parasitic infection, and language and motor delays. Approximately 20% of the total school population grades 1–8 were diagnosed with FASD (Table 1).

**Table 1:** FASD prevalence among students in Grades 1-8 at Atlantic First Nation School (1999–2000).

	**1999–2000**
**Total N**	**187**
***Rates of diagnosis***	
**FASD with SSF, or FAS**	
Male	7
Female	3
Total n (%)	10 (5.3)
**FASD without SFF, or pFASD/ARND**	
Male	21
Female	8
Total n (%)	29 (15.5)
FASD Total	39(20.8)
**SE (PAE Unknown)**	
Male	n/a
Female	n/a
Total n (%)	n/a
**PAE (At-Risk FASD)**	
Male	8
Female	1
Total n (%)	9 (4.8)
***Mean age at time of service access (in years)***	
FASD diagnosis	10.8
School support	10.8
Community support	None

ARND = Alcohol Related Neurodevelopmental Disorder; FAS = Fetal Alcohol Syndrome; FASD = Fetal Alcohol Spectrum Disorder; PAE = Prenatal Alcohol Exposure; pFAS = Partial Fetal Alcohol Syndrome; SE = Static Encephalopathy; SFF = Sentinel Facial Features; Dx= Diagnosis; n/a = not assessed. The initial screening process involved the entire school population Grades 1 through 8 being screened based on 1) teacher perception of children in their classes with multiple behavior and learning problems; and 2) semi-structured interviews with birth mothers/parents familiar with the child's developmental history. Following screening, a multi-disciplinary diagnostic process was used for FASD assessment by a team of health and service professionals that included both a physician and traditional healer.

### School and community-based resources

In response to the high rates of FASD, as well as other complex needs identified, the BCWC formed a Special Needs Sub-Committee and for the next few years met with representatives from the Band, the province and the federal government lobbying for funding to provide school and youth support services. If there was no funding available for an identified need, such as a school lunch program, members of the BCWC and school staff raised the money through agency and staff donations.

The BCWC also sponsored focus groups in the community funded by the Public Health Agency of Canada (PHAC) to define community priorities in relation to FASD (PHAC, 2006). Community members identified the need for a community-based model with a multi-pronged approach including FASD diagnosis, intervention and prevention. The BCWC received PHAC funding to develop a diagnostic team. The BCWC met with provincial health to leverage physician services as well as well as occupational and speech therapy assessment services. The Elsipogtog Health and Wellness Center (EHWC) donated office space and the services of a traditional healer. The Education division donated the services of the school psychologist. The EHWC donated services of nursing staff for a prevention program and the education division received funding from INAC for school interventions post-diagnosis. Based on the community input, collaborations and partnerships at all levels the ED Center opened in 2006 offering a comprehensive TES approach to FASD diagnosis, intervention and prevention.

**Figure 2:**
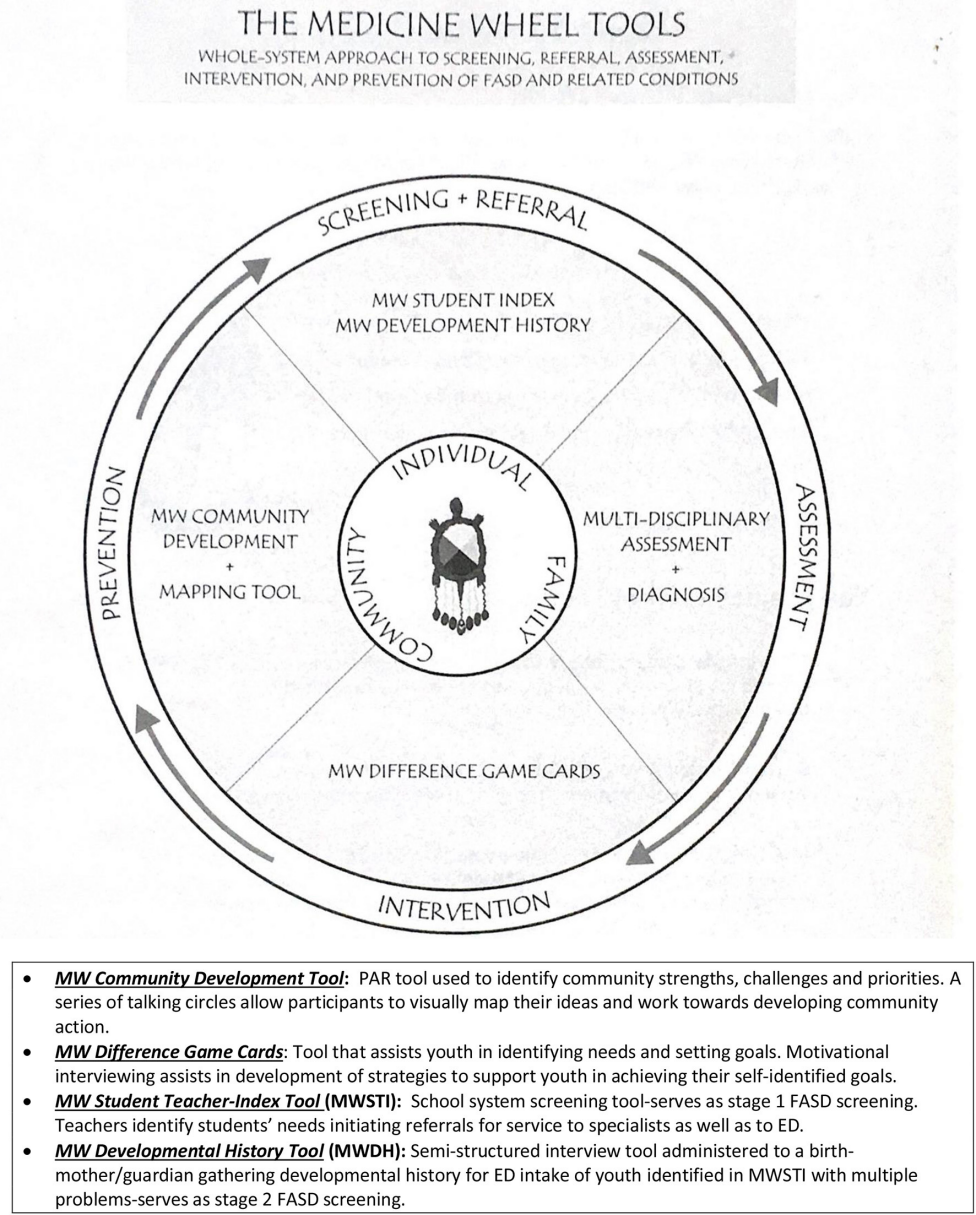
Medicine Wheel tools.

The MW Tools are used to guide and inform all aspects of FASD health service delivery at the ED. These tools were initially developed in the community but revised with input from a group of clinical researchers and Indigenous Elders chosen by PHAC for their expertise in the FASD MW tools field. This group ultimately choose the MW Tools for inclusion in a national FASD toolbox (Goh et al., 2008; CAPHC-Canadian Association of Pediatric Health Centres, 2012) (Figure 2).

## The Eastern Door Center model–key program elements

**Figure 3:**
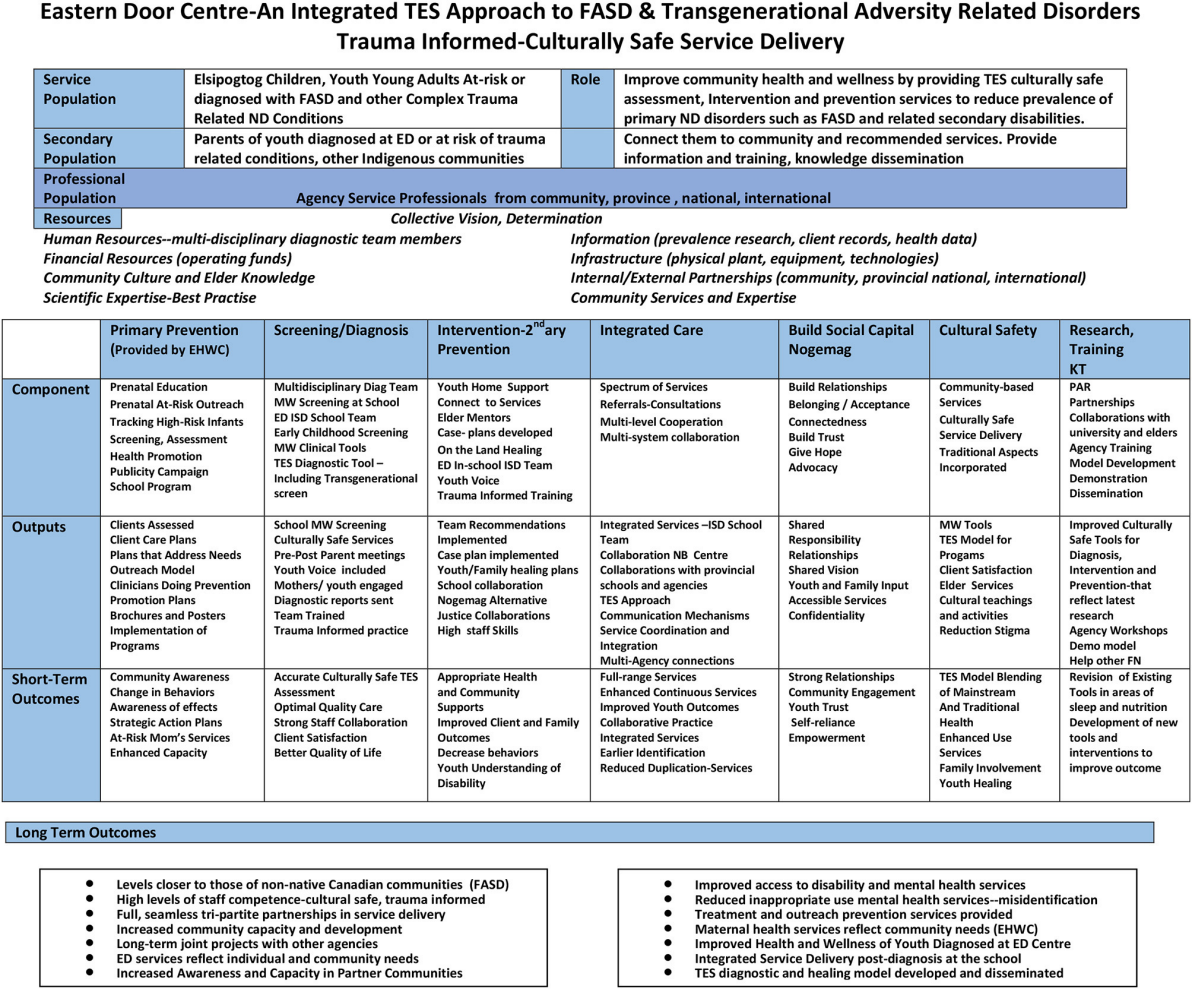
The Eastern Door model key elements.

The ED mandate is to reduce the prevalence of the primary FASD disability as well as associated secondary conditions through the delivery of culturally rooted trauma informed community based health services including diagnosis, intervention and prevention based on best-practice research (Figure 3).

### ED diagnostic team

ED uses a multi-disciplinary approach to diagnosis. The diagnostic team consists of: traditional healer, physician, nurse practitioner, psychologist, occupational therapist, physiotherapist, speech-language pathologist, resource coordinator, outreach workers and school counselor. The team was trained by Dr. Ted Rosalas and Dr. Sterling Clarren, both of whom were authors of diagnostic guidelines. The ED team uses both the Canadian FASD Guidelines and the 4-digit FASD diagnostic system (Astley, 2004; Cook et al., 2016). The 4-digit system allows for diagnosis of a (NDD) Neurodevelopmental Disorder when PAE is unknown (Astley, 2004). This diagnostic approach is more consistent with the perspective of the team traditional healer who looks at the dysfunction or “disordering” rather than the disorder. The ED team uses a TES process that includes a MW evaluation by the traditional healer as well as the standard diagnostic criteria (Clairmont, 2010).

**Figure 4:**
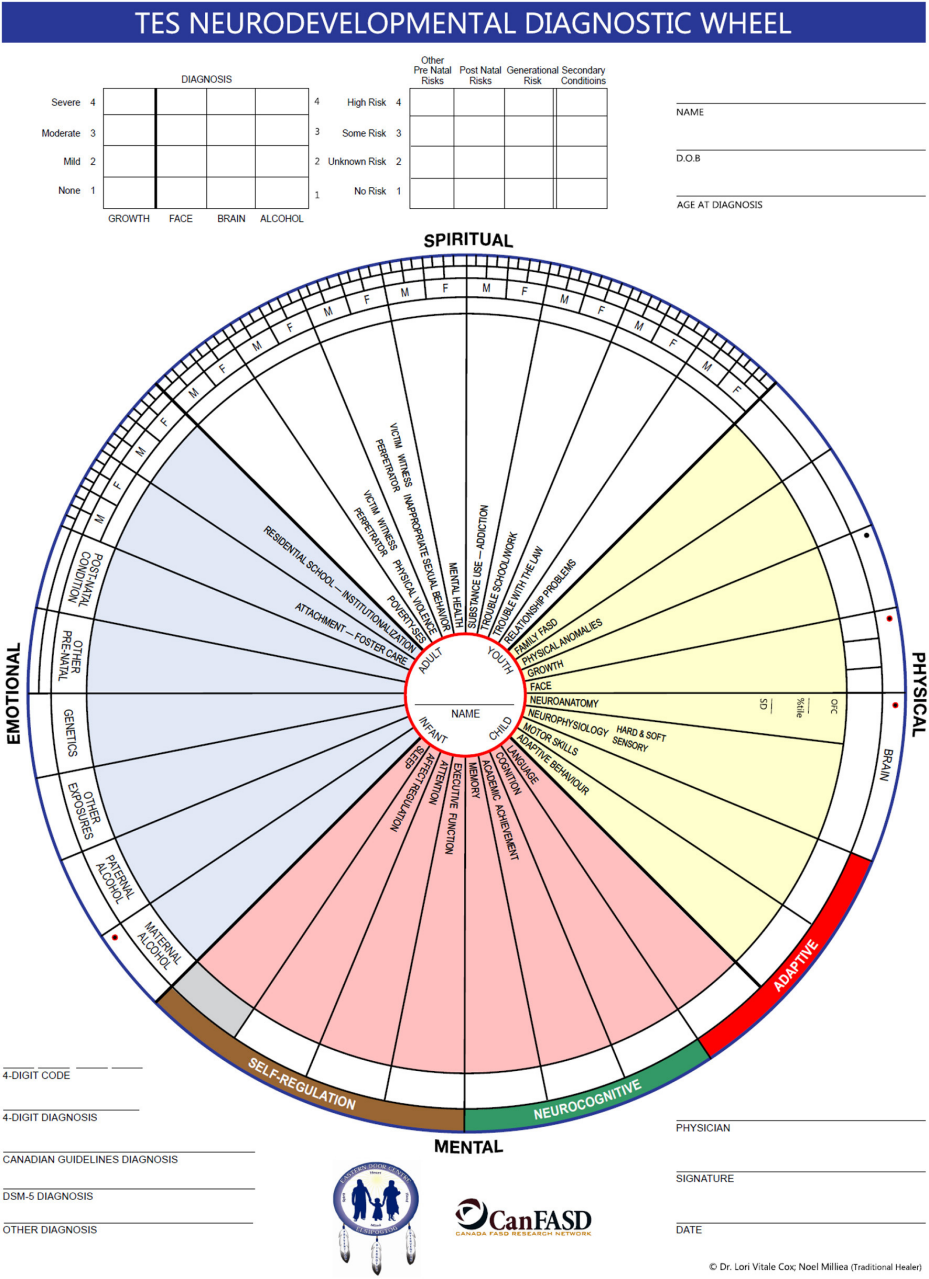
The Two-Eyed Seeing (TES) Neurodevelopmental Diagnostic Wheel.

Half of the professionals on the diagnostic team are Indigenous or married into the community; these team members are familiar with Mi'kmaq, the language of the community. A clinical tool, the TES Assessment Wheel, inspired by the team Elder is used during the diagnostic process. FASD is seen in the context of system adversity and transgenerational trauma rather than just the mother's behavior during pregnancy. Half of the TES wheel is a template to record the measurements needed for diagnosis using medically accepted diagnostic criteria. The other half looks at these conditions as life-time disorders, reflecting other factors that might contribute to how prenatal alcohol or drug exposure might be expressed in an individual, family and community system. There is consideration of residential schooling, paternal alcohol use, secondary conditions, and a generational family trauma component extending back three generations (Loock et al., 2020). From a TES perspective, improved outcomes and health depends upon a process of re-balancing the wheel and restoring relationships of the youth to self, family, the traditional community culture, and the natural world (Figure 4).

### Screening

The annual MW Teacher-Student Index Tool school screening is the primary source of referrals to the diagnostic clinic. The MW Teacher-Student Index Tool is now embedded in the schools Educational Information System (EIS). The ED team specialists also organize a pre-school screening for early identification of at-risk students who then can receive early intervention services that might ameliorate an FASD condition or other NDD before diagnosis. This early intervention also serves to distinguish children who are experiencing delays from those with more complex conditions that may require referral to ED.

### Intervention and prevention of adverse outcomes

#### Eastern Door-Integrated Service Delivery team (ED-ISD)

The ED-ISD team is made up of the same multi-disciplinary specialists who serve on the diagnostic team. Pre-diagnosis they analyze and prioritize the results of the annual MWSTI and provide an additional informal level of screening before referral for ED diagnosis. After referral the MWDH family interview is administered. This serves as the ED intake interview as well as providing second level screening for FASD and other NDD's in terms of obtaining maternal alcohol, drug and trauma disclosures and developmental history information needed for differential diagnosis. The ED-ISD team also provides the pre-K screening and early intervention services that can help ameliorate the effects of PAE. Post-diagnosis the ED-ISD team ensures delivery of specialist intervention services within the community school. They also assist in the design of Individual Education Plans (IEP) for ED youth in collaboration with teachers and parents.

#### ED Family Outreach (EDFO)

The role of EDFO is to support youth and families before and after diagnosis. Pre-diagnosis an EDFO worker interviews the birth mother and family members of referred youth with the MW Developmental History tool. This semi-structured interview tool allows the mother to digress and offer her feelings and thoughts. The interview is summarized by the EDFO worker and presented during clinic. The EDFO worker uses the MW Difference Game Cards to explore the youth's perspective, also presented during clinic. The ED is unique in terms of developing a formal tool to include youth's voice during the diagnostic process and to assist in case planning after diagnosis.

The EDFO worker sits with the parents at the post diagnostic meeting and debriefs with the parents and youth afterwards. EDOF works with parents and youth ensuring team recommendations are implemented by connecting families to the services that can support them. The EDFO also finds Elder mentors for each of the youth on their caseload. They help families and youth in practical ways supporting them in navigating service systems, serving as advocates until the youth and families can advocate for themselves. The philosophy of the program is that diagnosing one child and connecting their families to services may serve to prevent another FASD birth.

### Primary prevention

The Elsipogtog Health and Wellness Center now manages the prevention programs that ED developed and delivered from 2006 to 2015 in collaboration with them. These included: FASD awareness, a support group for young women who are involved with drugs and alcohol, and an outreach support program for high-risk women of child bearing years based on replication of Parent-Child Assistance Program (PCAP), an evidence based prevention model for substance using young women. In 2015, when the ED offices were moved from the EHWC to the Education administration building, staff at the EHWC began to manage and administer most of the community's FASD primary prevention programs.

### Nogemag Healing Lodge for youth

The Nogemag Healing Lodge for youth is an on-the land-healing program for high-risk youth. Many have been through the Eastern Door clinic. The program is presently funded through a collaborative initiative between groups in NB in the Acadian Peninsula, Inner-city Saint John, and other FN's. Nogemag offers after-school, holiday and summer programs. Elder mentoring, as well as hiking, canoeing and hunting programs, connect youth to the land and teach them traditional skills. The Nogemag summer camping program provides leadership training for older ED youth in high school who can apply for employment as counselors or assistant counselors. Nogemag was originally opened in 2001 as an alternative school for youth diagnosed with FASD who were in conflict with the law and suspended from school. An alternative school program still operates at the Nogemag site but is now managed by the community school.

### Training and research—National and International Collaborations, Training and Research

ED is a member of the Canada FASD Research Network (CanFASD). It participated in CanFASD's development of a Canadian database for FASD surveillance. ED collaborates nationally and internationally with American, Australian and New Zealand Indigenous community researchers and clinicians (CanFasd, 2022). ED is partnering on two CIHR research grants with the UBC Faculty of Medicine. It provides training for diverse groups and also serves as a demonstration model regionally and nationally. In 2015, the ED facilitated the development of the Elsipogtog Education Wellness Ethics Committee to guide research undertaken in the community. In 2021–22 a comparison was undertaken looking at FASD prevalence in 1999–2000 compared to twenty years later in 2021–22.

## Results and discussion–impacts in the community

### FASD diagnosis and prevalence rates

#### 1999–2000 estimated prevalence

Clinic records from the School Needs Assessment indicate that in 1999–2000 the estimated prevalence rate of FASD in school (grades 1–8) was 20.8%. 5.3% of the Grade 1–8 population were diagnosed with FAS (Table 2).

**Table 2:** Twenty-year comparison of prevalence of alcohol-related diagnoses and service access among students in Grades 1-8 at Elsipogtog First Nation School before and after implementation of culturally safe FASD service delivery.

	**1999–2000**	**2019–2020**
**Total N**	**187**	**233**
***Rates of diagnosis***		
**FASD with SSF, or FAS**		
Male	7	0
Female	3	0
Total n (%)	10 (5.3)	0 (0)
**FASD without SFF, or pFASD/ARND**		
Male	21	16
Female	8	8
Total n (%)	29 (15.5)	24 (10.3)
FASD Total	39 (20.8)	24 (10.3)
**SE (alcohol exposure unknown)** ^a^		
Male	-	10
Female	-	8
Total n (%)	-	18 (7.7)
**PAE (At-Risk FASD)**		
Male	8	1
Female	1	1
Total n (%)	9 (4.8)	2 (.8)
PAE/FASD Total	48 (25.7)	26 (11.2)
**Mean age at time of service access (in years)**		
FASD diagnosis	10.8	7.6
School support	10.8	5.0
Community support	None	7.6

ARND = Alcohol Related Neurodevelopmental Disorder; FAS = Fetal Alcohol Syndrome; FASD = Fetal Alcohol Spectrum Disorder; PAE = Prenatal Alcohol Exposure; pFAS = Partial Fetal Alcohol Syndrome; SE = Static Encephalopathy; SFF = Sentinel Facial Features. Over the years, the ED team has relied on both the 4-Digit Diagnostic Code (Astley, 2004) and the Canadian Guidelines (Chudley et al., 2005; Cook et al., 2016) to guide FASD assessment and diagnosis, thus diagnostic categories were merged across systems for presentation here.

The initial screening process involved the entire school population Grades 1 through 8 being screened based on 1) teacher perception of children in their classes with multiple behavior and learning problems; and 2) semi-structured interviews with birth mothers/parents familiar with the child's developmental history. Following screening, a multi-disciplinary diagnostic process was used for FASD assessment by a team of health and service professionals that included both a physician and traditional healer.

^a^Static encephalopathy was not considered during the 1999–2000 assessment year.

187 children in grades 1–8 (Total Population) were screened and referred for diagnosis if they had multiple problems with behavior and learning and their mothers disclosed PAE. The actual prevalence may have been higher since in the first clinic year the Pediatrician was not yet familiar with Alcohol Related Neurodevelopmental Disorder (ARND). Children with PAE who may have ARND were reported in the first year only as having PAE. This means the 4.8% of the cohort reported to have PAE may have had ARND; but they received no diagnosis related to FASD. In this first study there is also no data on the prevalence of Static Encephalopathy (SE)-PAE-unknown because this label was only established in 1999.

#### 2019–20 estimated prevalence

Twenty years later, the estimated FASD prevalence was reduced by approximately 50% (10.3 vs. 20.8%). No children were diagnosed with FAS (FASD with SFF). If the FASD prevalence had not been reduced than 20.8% of the school population (233) would be living with FASD. This would result in an estimated cost of C$1,152,000 per year If we use the recent FASD cost estimate of C$24,000 a year per individual (Greenmyer et al., 2018). The reduction in prevalence to 10.3% indicates an estimated savings of more than half a million dollars to the community, the province and the federal government (Table 2).

With ED practice there has been an increase in protective factors identified in the research: earlier diagnosis and implementation of disability accommodations in school and at home (Streissguth and Kanter, 1997). Children are referred and diagnosed earlier and interventions and supports in school and at home are implemented earlier.

Other notable results: ED's MW and TES clinical tools have been recognized nationally. The MW Teacher-Student Index Tool is available nationally on a First Nations Educational Information System. The MW Difference Game cards are used in mentoring programs in western Canada; the MW Community Development Tool has been used in FASD workshops in Yukon. Indigenous community teams nationally and regionally have visited ED for demonstration and training and invited ED staff to come to their communities to train their health and service professionals and to meet with community Elders. In one Cree community this involvement led to the development of their own FASD diagnostic team (Grand Council of the Cree-Eeyou Istchee, 2013).

One of the most significant accomplishments is ED's participation in 2009–10 in a Society of Obstetricians and Gynecologists of Canada (SOGC) expert roundtable that resulted in a revision to clinical guidelines on women's drinking that had previously suggested low-level drinking during pregnancy was safe (Carson et al., 2010). M'ikmaw traditional women's knowledge, frowned on alcohol use by young women during pregnancy (Marshall, 1999, 2008). This created a kind of cognitive dissonance in the community and reinforced a belief that FASD diagnosis in many cases was labeling. This distrust has largely given way with recognition of its positive effect on the wellbeing of youth and families and the growing involvement of community Elders in the ED process. One Elder in the community speaking at a Child Rights Summer Institute called ED “one of the most important healing programs in the community” (ISCROC, 2022).

### Reduction in stigma

FASD is not a culturally friendly concept because it holds within the name itself judgement regarding the mother's prenatal alcohol use as the cause of her child's disability. This judgement stigmatizes the mother who than is hesitant to disclose PAE. The ED widens the perspective in keeping with Elder knowledge in the community that FASD is a system disorder. The mothers drinking during pregnancy is one of many risk factors that result from transgenerational disordering. This perspective serves to reduce stigma.

A sample of parents who accessed clinic services from 2006 to 2010 were interviewed by an external evaluator. He reported parents rated their experience with diagnosis and family outreach positively giving ratings, respectively of 8 and 9 out of 10 (Clairmont, 2010). He also noted that the social stigma of FASD appeared to have diminished (Clairmont, 2010).

FASD diagnosis has come to be regarded less like a label because of the health and support that it offers that can change outcome. Some adults, diagnosed at the ED as youth, are successful and visible in the community; they do not hide their FASD diagnosis. They are married with their own children and working as fishermen or in the woods or in the community store. A few are back in the school as adults working as educational assistants helping youth with similar learning and behavior problems. Other Indigenous communities consider ED “an achievement” they would like to replicate in their own community (Networks for Indigenous Health Research (NIHR), 2018).

### Lessons learned

**Figure 5:**
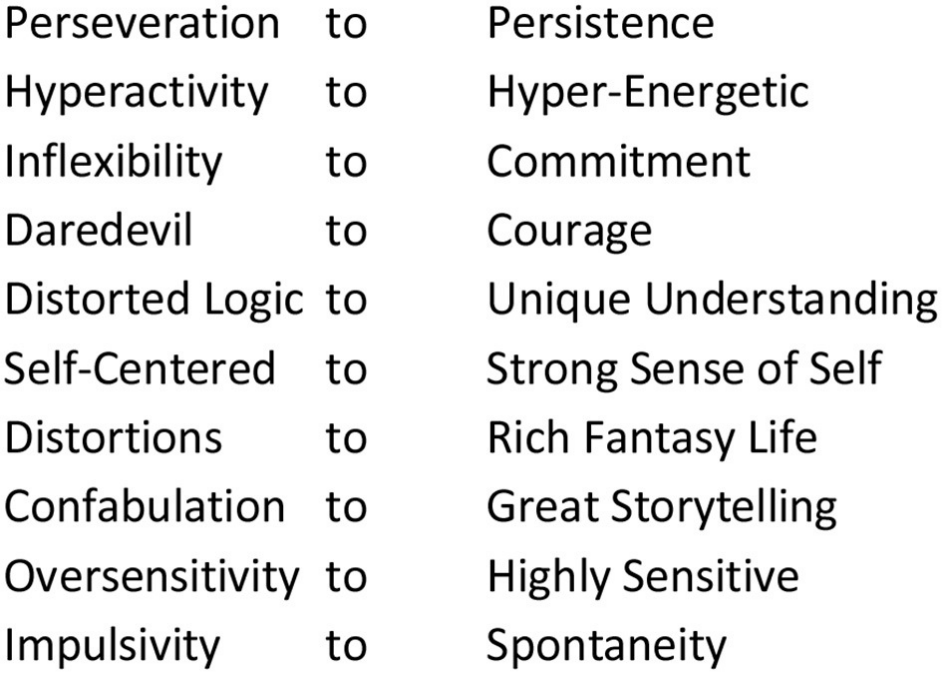
Weaknesses to strengths.

Listen to both Indigenous Elders and to Scientific Elders—let the perspectives of both guide clinical practice. For example, experienced FASD clinicians will build on particular areas of strengths in the profile of brain functioning in their post diagnostic recommendations. Traditional Elders have informed this strength-based practice by their insight that even the challenges of FASD can be perceived as “gifts from creator”—and they actually can become strengths—if the right supports and learning environment is provided. Don't try to change the child. Change the environment so that the child can learn to understand their challenges. In this way the challenges themselves can become gifts (Figure 5).

### Methodological issues–limitations

Without a control community we cannot say with certainty the reduction in FASD prevalence in the community is the direct result of implementation of the ED model. We can say that the model is a cost-effective approach to providing FASD health service delivery since the ED operates without core funding, sustaining itself through leveraging and integrating service delivery and through actively pursuing project funding.

While we know that there has been an increase in protective factors identified in the research and success of many youth we do not have any formal research data on the reduction of FASD related secondary disabilities.

The methods in the 2019–20 prevalence study included a level of informal screening before diagnostic referral by the ED-ISD team. This did not take place in the first study. Although this methodology is different it would serve to decrease false positives in the first stage screening with the MWSTI.

In 2019–20, 7.7% of the children in the grade 1–8 cohort fit the 4-Digit Diagnostic criteria for Static Encephalopathy—PAE Unknown. In 1999–2000, however, there is no data available to determine the prevalence of this condition for comparison. This has no effect on FASD prevalence estimates but does raise related questions. Reports indicate that there has been a decrease in the use of alcohol during pregnancy but a sharp rise in the use of opiates. Clairmont (2010). This pattern is likely an indication of the on-going adversity in the community leading to young women replacing the use of alcohol with other more addictive substances. Mothers of children diagnosed with SE-PAE-unknown in 2019–20 disclosed they had used a variety of drugs when pregnant including nicotine, methadone, cocaine, cannabis, oxycodone and diazepam. Are they also using minimal quantities of alcohol they do not remember or even report? Is there a synergistic effect of multi-substance prenatal exposure? More investigation and discussion is needed that is beyond the scope of this paper.

The ED is unable to provide the kind of comprehensive step by step MW approach required for transgenerational healing. The ED is unable to provide FASD support services to youth after 18 once they leave school. Without support some ED youth turn to drugs and alcohol and get in conflict with the law. The children of these ED clients then become ED clients themselves. The health and wellbeing of ED youth is related to family and community system stability. Yet the ED does not provide the level of intervention services that the mothers and families of the ED youth require. So the generational pattern continues. The ED is unable to provide connections to the services families and young adults living with FASD need because these services do not exist in the Indigenous community system. Indigenous Services Canada does not fund FASD diagnostic services nor a comprehensive community based approach to FASD service delivery. This is considered to be a provincial responsibility and despite the Calls to Action of the Truth Reconciliation Commission of Canada (TRC) (2015) FASD service delivery continues to be almost non-existent for young Indigenous adults who come into conflict with the law, because their disability is undiagnosed and unsupported in the community system. System adversities continue to disadvantage Indigenous people. Unless these adversities are dealt with health inequalities like FASD will continue to be the result of health inequities (Adelson, 2005).

## Conclusion

Despite the limitations of the ED model, it works on many levels through collaborative practice to improve and restore relationships and connections of the youth to themselves, their families and their community. One of the mothers who accessed FASD services in the community speaks of her experience (Cox, 2015).

When she was a baby I didn't understand……..she would get angry (and) bang her head on the floor…..like constantly…..she didn't know how to say how she was feeling……At first I dreaded going ….didn't know….didn't want to know….but I put all those feelings aside…..I said this is for her…… She (EDO) asked me a lot of questions and I just answered them in my honest way as best as I could……but she helped me figure out a lot of stuff in my head…just talking to her it was a relief….…that it had a name….the doctor told me that this is what it is and then what they were going to do….every word after that…it was lifting my spirits and then things started going more easier….…and she started to learn and when she first started reading a book I cried…I never thought she would read a book……and I was so happy…when she graduated high school—all that, because of that, the diagnosis, it made my life easier—Thank you ED.

Elder Albert Marshall noted that a TES approach is a “way of doing things” a collaborative practice (Marshall et al., 2009). Mi'kmaq Elder Charles Labrador said “Go into the forest and see birch, maple and pine—look underground and all those trees are holding hands. We as people must do the same” (Iwama, 2009).

## Data availability statement

The original contributions presented in the study are included in the article/supplementary material, further inquiries can be directed to the corresponding author.

## Ethics statement

The studies involving human participants were reviewed and approved by UBC Ethics Committee Elsipogtog Education Wellness Ethics Committee. Written informed consent to participate in this study was provided by the participants' legal guardian/next of kin.

## Author contributions

LC is the main researcher who collected and analyzed the data. Co-authors Ivan Augustine and Eva Sock do not have university affiliation so I was unable to include them but they should be. They have collaborated and been involved in the models development. Eva was the previous Director of the ED Center and she came up with the name Eastern Door.
